# Magnesium level correlation with clinical status and quality of life in women with hormone related conditions and pregnancy based on real world data

**DOI:** 10.1038/s41598-021-85156-y

**Published:** 2021-03-11

**Authors:** Svetlana Orlova, Galina Dikke, Gisele Pickering, Eliso Djobava, Sofya Konchits, Kirill Starostin

**Affiliations:** 1 Department of Dietetics and Clinical Nutritiology of Continuing Medical Education, Medical Institute, RUDN University, Moscow, Russia; 2 Department of Obstetrics and Gynecology with a Course of Reproductive Medicine, The Academy of Medical Education F.I. Inozemtsev, Saint-Petersburg, Russia; 3 Department of Clinical Pharmacology Inserm CIC 1405, University Hospital, Clermont-Ferrand, France; 4 Inserm 1107 Fundamental and Clinical Pharmacology of Pain, Medical Faculty, Clermont-Ferrand, France; 5 Department of Obstetrics and Gynecology, Russian National Research Medical University, Moscow, Russia; 6 Department of Medical Affairs, Sanofi, Business Center “Summit”, Tverskaya 22, Moscow, Russia 125009

**Keywords:** Nutrition, Quality of life, Therapeutics

## Abstract

This study was aimed to assess the effectiveness of magnesium (Mg)-vitamin B 6 replenishment and its correlation with clinical status in pregnant women (PW), and quality of life in women with hormone-related conditions (HRCW) and hypomagnesemia (HME). Data collected in four observational studies were pooled and analysed. All women received Mg supplementation for 4 weeks. The proportion of women with normalized Mg level, and the correlation between serum Mg dynamics and number of symptoms/complaints (PW) or changes in World Health Organization quality of life questionnaire scores (WHOQOL; HRCW) were evaluated. 869 PW and 957 HRCW were included in the study. Normalization of serum Mg level to ≥ 0.66 mmol/L occurred in 92.1% of PW and 78.4% of HRCW, and to ≥ 0.8 mmol/L in 73.8% and 58.9%, respectively. Mg normalization was accompanied by a median decrease of 1 symptom and 1 complaint in PW. Serum Mg level increase by 0.1 mmol/L was associated to significant changes in the WHOQOL scores in HRCW. Treatment of HME with the Mg for approximately 4 weeks provided a high response rate of Mg serum level, was associated with an improvement in symptom severity and complaints in PW, and WHOQOL score in HRCW. A 0.8 mmol/L cut-off appeared to be more relevant in terms of patient-reported outcomes.

## Introduction

Magnesium (Mg) is one of the most prevalent cations in the body and plays numerous vital physiological functions^1–3^. It is essential for the activity of hundreds of enzymes encompassing 80% of known metabolic functions^4, 5^. Therefore, disturbances in Mg homeostasis may be involved in a wide variety of pathological processes.

Despite the importance of Mg for human health, 60% of people do not meet the recommended daily intake (320 mg/day for women; 420 mg/day for men)^4^. Common causes of Mg deficiency include inadequate dietary intake or gastrointestinal absorption, loss through the gastrointestinal or renal system, and pregnancy, where there is an increased requirement for Mg^6^. Mg deficiency is more frequent in women than men and specific Mg functions in women’s health are well recognized^6^, like premenstrual syndrome, osteoporosis, cancer and menopause^7^.

In healthy subjects, total serum Mg levels range between 0.7 and 1.0 mmol/L^8, 9^. Hypomagnesemia (HME) is defined as lower serum Mg levels but its reference values can vary greatly across different countries^10^. Currently in the Russian Federations, total serum Mg < 0.66 mmol/L is considered as the common value indicating Mg deficiency in adults^11^, but total serum Mg < 0.75 mmol/L is also frequently used in basic laboratory research^10^. Additionally, several recent publications based on an epidemiological approach suggested an optimal HME cut-off value of < 0.85 mmol/L^12, 13^.

Four large observational studies were conducted from 2012–2016 across the Russian Federation to study the prevalence and clinical management of Mg deficiency in pregnant women (MAGIC, MAGIC2)^14, 15^ and in women with hormone-related conditions (MAGYN, MAGYN2)^16, 17^ using the Mg deficiency questionnaire (MDQ) and laboratory tests. A secondary analysis of pooled data collected from all four studies was performed. Here, the effects of Mg supplementation on the quality of life (QoL) of pregnant women and women with hormone-related conditions are presented. Additionally secondary analyses included the development of a modified MDQ to ease the estimation of the prevalence of Mg deficiency, and the epidemiological assessment of risk factors and comorbidities associated with Mg deficiency were investigated in the studied populations. The results of these secondary analyses have been published elsewhere^18, 19^.

## Methods

### Study population and treatment

Pregnant women with clinical evidence of Mg deficiency related to pregnancy were recruited in the MAGIC (N = 1130)^14^ and MAGIC2 (N = 2117)^15^ observational studies. Women suffering Mg deficiency due to any other concomitant known cause were excluded. A total of 9168 (MAGYN)^16^ and 11,424 (MAGYN2)^17^ women with hormone-related conditions who had HME were enrolled while attending outpatient clinics. Women aged 18–60 years and receiving hormonal contraception or hormone replacement therapy (HRT), with premenstrual syndrome (PMS), climacteric syndrome without HRT, or osteoporosis, were included, as well as women of reproductive age with other hormonal conditions such as endometriosis, polycystic ovarian disease, uterine leiomyoma, algodysmenorrhea, or endometrial hyperplastic processes. Women suffering severe conditions that could have potentially hindered participation or were receiving Mg supplementation at the time of enrollment, were excluded^14–16^.

All primary observational studies (MAGIC, MAGIC2, MAGYN, MAGYN2) were approved by independent ethical committee (“The Independent Multidisciplinary Committee on Ethical Review of Clinical Trials”, 125468, Russia, Moscow, 51 Leningradskiy ave.). This analysis including all methods was carried out in accordance with all relevant guidelines and regulations applicable in Russian Federation.

All women who received a combination of Mg and vitamin B_6_ (MgB_6_) as Magne B_6_ or Magne B_6_ Forte (Sanofi) for the treatment of HME were included in the analysis. Serum Mg concentrations were measured at Visit 1 and about 4 weeks after receiving MgB_6_ supplementation (Visit 2) using 0.66 mmol/L and 0.8 mmol/L serum Mg level as cut-off values. All women meeting the eligibility criteria of the observational studies who also had results of the total serum Mg tests at Visit 2 were included, with the exception of those having missing, conflicting, or outlier data (exclusion was performed separately for each objective).

### Study objectives

The focus of this secondary analysis was to evaluate:

1. The effectiveness of Mg replenishment in pregnant women and women with hormone-related conditions. Depending on the cut-off values used, the target normalization of total serum Mg was defined as ≥ 0.66 mmol/L or ≥ 0.8 mmol/L.
2. The correlation of the change in the total serum Mg level (ΔMg_V2−V1_) with the change in the number of complaints, threatened miscarriage symptoms (ΔN_comp/symp_) and pregnancy course from Visit 1 to Visit 2.
3. The correlation of ΔMg_V2−V1_ with the changes in the QoL from Visit 1 to Visit 2 in the group of women with hormone-related conditions.
4. The factors independently contributing to the total serum Mg normalization in both study cohorts.

### Analysis

The effectiveness of Mg replenishment in both cohorts of pregnant women and women with hormone-related conditions was assessed by the proportion of women who reached total serum Mg normalization after receiving supplementation.

**Figure 1:**
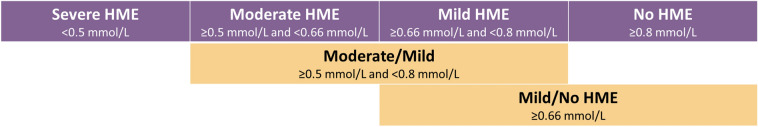
Subgroup categories and relative serum Mg concentration ranges. *HME* hypomagnesemia.

In the cohort of pregnant women who reached normal serum Mg levels after supplementation, ΔN_comp/symp_ was defined as the mean difference between Visit 1 and Visit 2 in the number of complaints and in the number of threatened miscarriage symptoms. In the pregnant women cohort, the relationship between ΔMg_V2−V1_ and ΔN_comp/symp_ was analysed using Pearson’s correlation. A linear regression model was conducted to assess the impact of ΔMg_V2−V1_ (normalized by 0.1 mmol/L; ΔMg_0.1_) on ΔN_comp/symp_. For this second analysis women were distributed based on serum Mg level at Visit 1 into *severe HME*, *moderate* HME, *mild HME*, and *no HME* subgroups. Subgroups and their relative serum Mg ranges are summarized in Fig. 1.

Changes in the QoL of women with hormone-related conditions were assessed by using the short version of the World Health Organization quality of life questionnaire (WHOQOL-BREF)^20^. The WHOQOL-BREF comprised 26 items. The first two questions were general (“How would you rate your quality life?” and “How satisfied are you with your health?”), while the remaining 24 questions were grouped in 4 domains which measured the following aspects: physical health, psychological health, social relationships, and environment (see details in Supplementary Information 1.1). The mean difference between Visit 1 and Visit 2 in the total WHOQOL-BREF score (Δ_WHOOL-BREF_) among the group of women who reached normal serum Mg level was assessed. The relationship between ΔMg_V2−V1_ and changes in QoL from Visit 1 to Visit 2 was evaluated in the cohort of women with hormone-related conditions using Pearson’s correlation coefficient *r* between ΔMg_V2−V1_ and Δ_WHOOL-BREF,_ and a linear regression model of Δ_WHOOL-BREF_ as the function of ΔMg_01_.The same subgroups as the cohort of pregnant women were considered (i.e. severe HME, moderate HME, mild HME, and no HME).

Factors that were independently predictive of the normalization of serum Mg concentration (at ≥ 0.8 mmol/L and ≥ 0.66 mmol/L) in response to MgB_6_ supplementation were investigated in both cohorts of women, pregnant women and women with hormone-related conditions.

### Statistical methods

The analysis population comprised all women who had HME at Visit 1 (either by serum Mg < 0.66 mmol/L or < 0.8 mmol/L cut-off values), who received MgB_6_ supplementations and had the results of serum Mg test (and WHOQOL-BREF scores for the women with hormone-related conditions) at Visit 2. During the analysis, women were allocated in different subgroups depending on the serum Mg results at Visit 1. According to the scientific literature, two reference values are commonly used in clinical practice to define serum magnesium deficiency: 0.66 mmol/L and 0.8 mmol/L^13, 21^. In this analysis, serum Mg values below 0.5 mmol/L were used to define a status of severe HME; values ranging from 0.5 mmol/L to 0.66 mmol/L or 0.8 mmol/L, were used to define moderate and moderate/mild HME, respectively; Mg values ranging between 0.66–0.8 mmol/l were referred to as mild HME; and, lastly, values above 0.8 mmol/L were considered as no HME (Fig. 1).

Characterization of the groups was performed using descriptive statistics, including calculation of mean, standard deviation (SD), median, lower and upper quartiles (Q1; Q3), and proportions (where appropriate). Differences in Mg levels between the analysed groups were investigated using the chi-square test (for categorical variables comparing responding rate between subgroups), unpaired *t* test (in case of normal distribution), and non-parametric tests (Wilcoxon signed-rank test to analyse whether serum Mg level changed significantly from baseline to week 4). Correlation analysis with linear regression was used to identify interactions between changes in serum Mg level and changes in QoL, symptoms and clinical or laboratory tests in pregnant women and women with hormone-related conditions. The factors that independently predicted normalization of serum Mg concentration were identified using univariate logistic regression analysis. No sensitivity analysis was performed.

### Ethics approval

All primary observational studies (MAGIC, MAGIC2, MAGYN, MAGYN2) were approved by independent ethical committee (“The Independent Multidisciplinary Committee on Ethical Review of Clinical Trials”, 125,468, Russia, Moscow, 51 Leningradskiy ave.) and were conducted in accordance with the principles of good clinical practice and the laws of the Russian Federation.

### Consent to participate

Informed consent has been collected from all participants of the primary observational studies (MAGIC, MAGIC2, MAGYN, MAGYN2) in accordance with the principles of good clinical practice and the laws of the Russian Federation.

### Consent for publication

All authors agreed to publish this manuscript.

## Results

### Baseline and demographic characteristics

**Figure 2:**
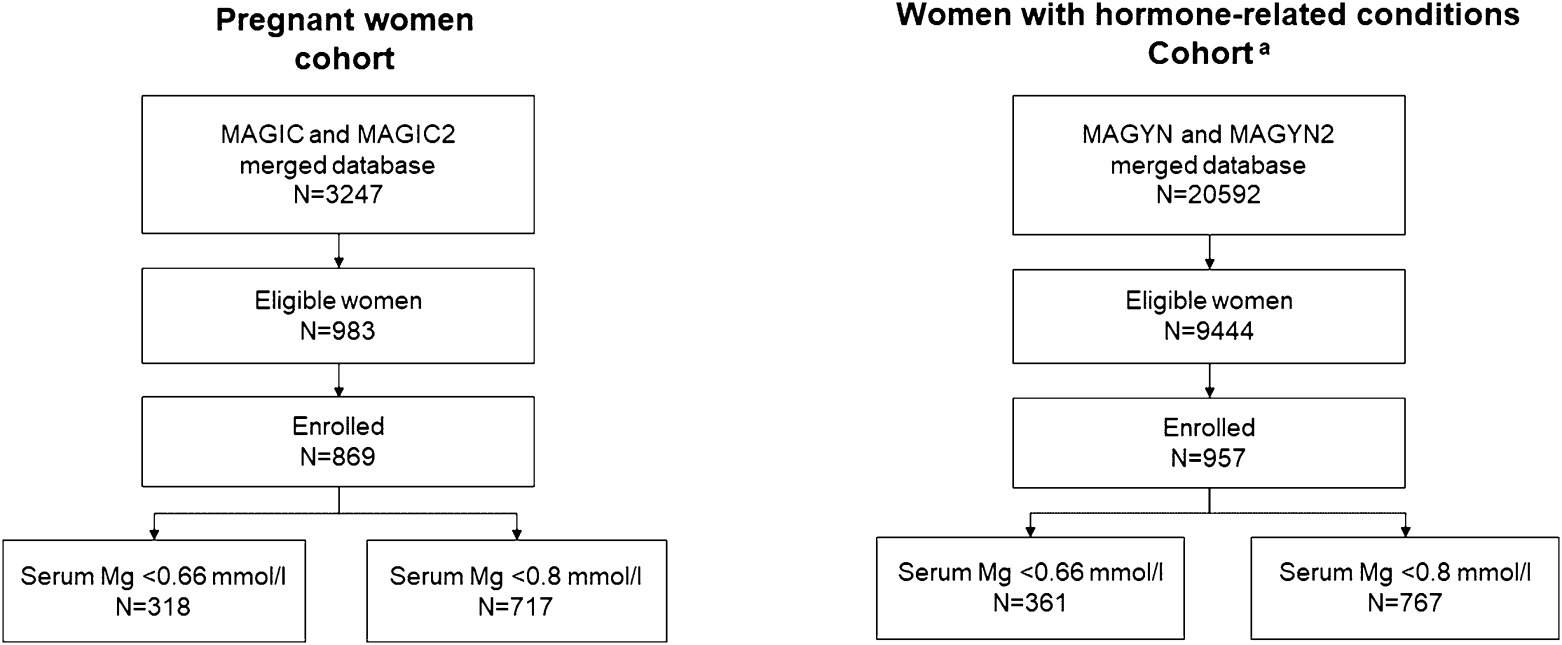
Study population. ^a^Severe HME (n = 50) cases were excluded from subgroup analysis of women with hormone-related conditions cohort. *Mg* magnesium.

In total, 983 women in the pregnant women cohort and 9444 women in the hormone-related conditions cohort had HME and were eligible for the study; of those, 869 and 957 were included in the analysis, respectively, and allocated in serum Mg subgroups (≥ 0.66 mmol/L and ≥ 0.8 mmol/L) based on the results at Visit 1 (Fig. 2). Women in the pregnant women cohort had a median age of 28 years (range 18–52) and mean (SD) serum Mg level of 0.71 (0.13) mmol/L (range 0.12–1.92) at Visit 1, while women in the hormone-related conditions cohort had a median age of 44 years (range 18–60) and mean (SD) serum Mg level of 0.77 (0.20) mmol/L (range 0.08–4.08). The prevalence of Mg deficiency assessed by serum blood levels was 34.0% in pregnant women and 24.1% in women with hormone-related conditions when using < 0.66 mmol/L as the cut-off value for the diagnosis; the cut-off of < 0.8 mmol/L provided a more than two times higher prevalence of HME among women, 78.9% and 54.8%, respectively. Hypermagnesemia (total serum Mg > 1.2 mmol/L) was rare in both cohorts: 0.5% in pregnant women and 1.9% in women with hormone-related conditions.

### Proportion of women who reached the target serum Mg level in both cohorts

**Figure 3:**
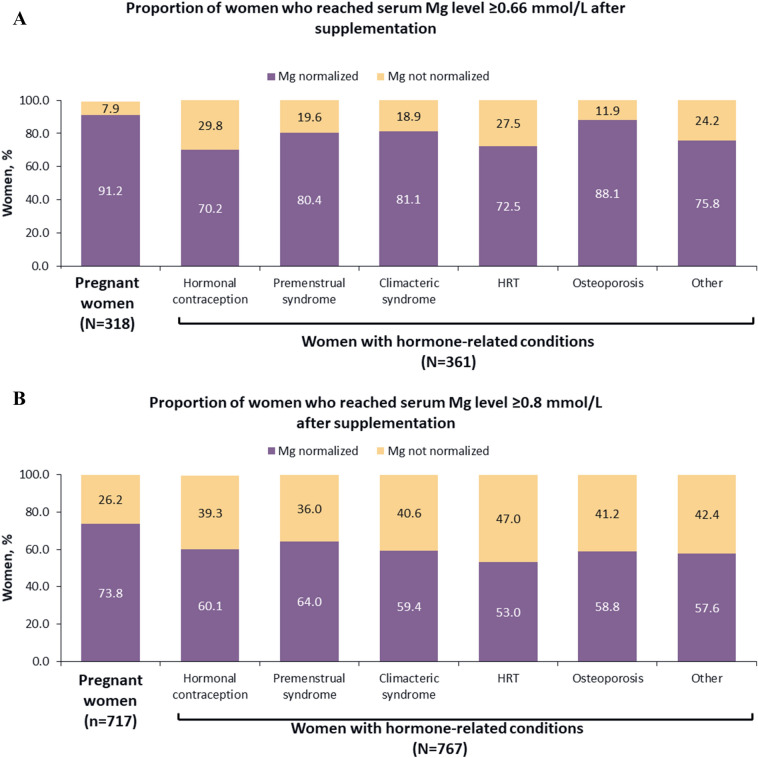
Proportion of women who had HME at Visit 1 and who reached target Mg serum level within 4 weeks after supplementation; HME was defined using (**A**) < 0.66 mmol/L or (**B**) < 0.8 mmol/L. Other, endometriosis; polycystic ovarian disease; uterine leiomyoma; algodismenorrhea; endometrial hyperplastic processes. *HME* hypomagnesemia; *HRT* hormone replacement therapy; *Mg* magnesium.

After taking MgB_6_ supplementation for four weeks, normalization of serum Mg level to ≥ 0.66 mmol/L occurred in 92.1% of pregnant women and 78.4% of women with hormone-related conditions. The corresponding proportions for serum Mg level ≥ 0.8 mmol/L were 73.8% and 58.9%, respectively (Fig. 3). Among women with hormone-related conditions, the best response rate was recorded in women with osteoporosis (88.1%) for the cut-off < 0.66 mmol/L and in women with premenstrual syndrome (64.0%) for the cut-off < 0.8 mmol/L. Response rate distribution among subgroups of women with hormone-related conditions was not significant (chi square test). Changes in serum Mg concentration from V1 to Visit 2 (ΔMg_V2–V1_) are shown in Table 1.

**Table 1:** Changes in serum Mg levels in pregnant women and women with hormone-related conditions from baseline to week 4 (ΔMg_V2−V1_).

	Pregnant women	Hormonal contraception	Premenstrual syndrome	Climacteric syndrome	HRT	Osteoporosis	Other
**Mg level at baseline < 0.66 mmol/L, N**	318	39	37	40	38	38	43
Mean ΔMg_V2−V1_,SD	0.342 (0.432)	0.140 (0.138)	0.122 (0.105)	0.187 (0.167)	0.173 (0.167)	0.222 (0.174)	0.128 (0.110)
Median ΔMg_V2−V1_, Q1, Q3	0.370 (0.190, 0.420)	0.120 (0.000, 0.200)	0.110 (0.040, 0.190)	0.135 (0.055, 0.280)	0.120 (0.050, 0.250)	0.240 (0.070, 0.310)	0.110 (0.020, 0.200)
*p* value	< 0.0001	< 0.0001	< 0.0001	< 0.0001	< 0.0001	< 0.0001	< 0.0001
**Mg level at baseline < 0.8 mmol/L, N**	717	81	66	60	60	66	84
Mean ΔMg_V2−V1_,SD	0.255 (0.320)	0.118 (0.155)	0.107 (0.105)	0.162 (0.155)	0.143 (0.153)	0.159 (0.166)	0.094 (0.122)
Median ΔMg_V2−V1_, Q1, Q3	0.250 (0.100, 0.390)	0.100 (0.030, 0.180)	0.095 (0.010, 0.180)	0.130 (0.055, 0.200)	0.095 (0.035, 0.200)	0.100 (0.030, 0.250)	0.075 (0.035, 0.145)
*p* value	< 0.0001	< 0.0001	< 0.0001	< 0.0001	< 0.0001	< 0.0001	< 0.0001

ΔMg_V2−V1_, difference between Visit 1 and Visit 2 in serum Mg concentration; *HRT* hormone replacement therapy; *Mg* magnesium; *N* number of women; *Q* quartile; *SD* standard deviation; *p* values reflect whether serum Mg level changed significantly from baseline to week 4 (Wilcoxon signed-rank test).

### Correlation of the change in total serum Mg level with the number of complaints and symptoms of threatened miscarriage in pregnant women

The increase in total serum Mg level was related to a decrease in the number of complaints (comprising of oedema, low abdomen heaviness, bleeding, tetany, pain in the area of the wide ligaments of the uterus and symphysis) as well as threatened miscarriage symptoms (including myometrium hypertonus, chorion detachment, preeclampsia, placental insufficiency). As shown in Table 2, the normalization of the total serum Mg level to ≥ 0.66 mmol/L and to ≥ 0.8 mmol/L was accompanied by a median decrease in the number of symptoms of threatened miscarriage and complaints equal to 1 symptom and 1 complaint for both cut-off values. A negative, although weak, correlation was observed between ΔMg_V2−V1_ and the changes in the number of symptoms (r =  − 0.1253; p < 0.001) and complaints (r =  − 0.1074; p = 0.002). Linear regression analysis showed that an increase of 0.1 mmol/L in total serum Mg level was associated with a statistically significant decrease in the number of complaints and threatened miscarriage symptoms in the moderate/mild HME subgroup (N = 698) and in the mild/no HME subgroup (N = 551), but not in the moderate HME subgroup (Table 3). Additionally, combined subgroups of women with any HME were analysed, and statistically significant negative effects of 0.1 mmol/L increase in total serum Mg level were found only in women with severe and moderate/mild HME (N = 717; Table 3), but not moderate HME.

**Table 2:** Changes in the number of complaints and threatened miscarriage symptoms in pregnant women with symptoms of Mg deficiency in whom total serum Mg level was normalized.

Parameter	N	Mean ΔN_V2−V1_	SD	Median ΔN_V2−V1_	Q1	Q3
**Normalization of Mg level from < 0.66 mmol/L to ≥ 0.66 mmol/L (N = 318)**						
Complaints number	293	− 1.167	1.061	− 1	− 2	0
Symptoms number	293	− 0.625	0.760	− 1	− 1	0
**Normalization of Mg level from < 0.8 mmol/L to ≥ 0.8 mmol/L (N = 717)**						
Complaints number	529	− 1.170	0.972	− 1	− 2	0
Symptoms number	529	− 0.637	0.742	− 1	− 1	0

ΔN_V2−V1,_ difference between Visit 1 and Visit 2 in the number of symptoms or complaints reported; *Mg* magnesium; *N* number of women; *Q* quartile; *SD* standard deviation.

**Table 3:** Linear regression results of ΔN_comp/symp_ as function of the difference in the total serum Mg level between Visit 1 and Visit 2 normalized by 0.1 mmol/L (ΔMg_0.1_) in the severe, mild and no HME subgroups.

HME subgroup	N	Parameter	ΔN_comp/symp_	SE	*p* value
Severe HME	19	Complaints	− 0.347	0.175	0.0639
		Symptoms	− 0.106	0.093	0.2718
Moderate HME	299	Complaints	− 0.021	0.034	0.5343
		Symptoms	− 0.022	0.025	0.3885
Moderate/mild HME	698	Complaints	** − 0.052**	**0.021**	**0.0148**
		Symptoms	** − 0.051**	**0.016**	**0.0012**
Severe + moderate/mild HME	717	Complaints	** − 0.073**	**0.021**	**0.0005**
		Symptoms	** − 0.058**	**0.015**	**0.0001**
Mild/no HME	551	Complaints	** − 0.070**	**0.024**	**0.0039**
		Symptoms	** − 0.052**	**0.019**	**0.0056**
No HME	152	Complaints	− 0.078	0.042	0.0697
		Symptoms	− 0.002	0.036	0.9645

Severe HME, serum Mg < 0.5 mmol/l; Moderate HME, serum Mg ≥ 0.5 mmol/L and < 0.66 mmol/L; Moderate/Mild HME, serum Mg ≥ 0.5 mmol/L and < 0.8 mmol/L; Mild/No HME, serum Mg ≥ 0.66 mmol/L and < 0.8 mmol/L; No HME, serum Mg ≥ 0.8 mmol/L. HME, hypomagnesemia; ΔN_comp/symp,_ mean difference in the number of complaints, threatened miscarriage symptoms; ΔMg_0.1_, difference in the total serum Mg level between Visit 1 and Visit 2 normalized by 0.1 mmol/L; *Mg* magnesium; *N* number of women; *SE* standard error; *p* values reflect whether serum Mg level change is significantly and linearly related to lower number of complaints/symptoms.

Significant p values (<0.05) are presented in bold.

### Correlation of the changes in total serum Mg level with the changes in QoL in women with hormone-related conditions

The normalization of total serum Mg level to ≥ 0.66 mmol/L and ≥ 0.8 mmol was accompanied by an increase in every WHOQOL-BREF domain score (Table 4). The correlation between ΔMg_V2−V1_ and Δ_WHOOL-BREF_ was weak but significant, as shown in Table 5. Based on Mg data collected at Visit 1, the linear regression analysis was conducted on women in the moderate (N = 361), moderate/mild (N = 767) and no HME (N = 546) subgroups. An increase of 0.1 mmol/L in total serum Mg level was found to have a statistically significant positive effect on Δ_WHOOL-BREF_ in all four domains and general Question 1 and 2 only in the subgroup of moderate/mild women (serum Mg < 0.8 mmol/L; Table 6). Women with severe HME were not included in the linear regression analysis due to limited sample size (N = 50), inadequate to obtain reliable results. No statistically significant difference was found in the QoL of the subgroup with serum Mg level ≥ 0.8 mmol/L (no HME).

**Table 4:** Changes in WHOQOL-BREF scores in women with hormone-related conditions in whom total serum Mg level was normalized.

Parameter	N	Median Δ_WHOQOL-BREF_	Q1	Q3
**Normalization of Mg level from < 0.66 mmol/L to ≥ 0.66 mmol/L**				
Domain 1	299	6	4	10
Domain 2	297	5	2	7
Domain 3	304	2	1	4
Domain 4	301	4	1	7
**Normalization of Mg level from < 0.8 mmol/L to ≥ 0.8 mmol/L**				
Domain 1	449	6	3	8
Domain 2	451	4	2	6
Domain 3	458	2	0	3
Domain 4	454	3	1	6

*Mg* magnesium; *Q* quartile; *WHOQOL-BREF* World Health Organization quality of life questionnaire; Δ_WHOQOL-BREF_, difference between Visit 1 and Visit 2 in the total WHOQOL-BREF.

**Table 5:** Correlation coefficients r between changes in WHOQOL-BREF scores and the difference in Mg level in women with hormone-related conditions (N = 957).

	Question 1	Question 2	Domain 1	Domain 2	Domain 3	Domain 4
N	955	957	939	941	953	949
r	0.1218	0.0879	0.1814	0.1689	0.1584	0.1567
*p* value	< 0.001	0.007	< 0.001	< 0.001	< 0.001	< 0.001

*Mg* magnesium; *WHOQOL*-*BREF* World Health Organization quality of life questionnaire; *p* values reflect whether Pearson’s coefficient of correlation is statistically significant between changes in WHOQOL-BREF question/domain scores and the difference in serum Mg level.

**Table 6:** Linear regression results of Δ_WHOQOL−BREF_ as function of ΔMg_0.1_

HME subgroup	N	Domain	Δ_WHOQOL−BREF_	SE	*p* value
Moderate HME	361	Question 1	**0.07**	**0.03**	**0.0364**
		Question 2	0.02	0.03	0.4564
		Domain 1	**0.46**	**0.17**	**0.0072**
		Domain 2	**0.36**	**0.14**	**0.0092**
		Domain 3	**0.25**	**0.09**	**0.0071**
		Domain 4	**0.41**	**0.19**	**0.0286**
Moderate/mild HME	767	Question 1	**0.09**	**0.02**	**0.0000**
		Question 2	**0.06**	**0.02**	**0.0060**
		Domain 1	**0,64**	**0,12**	**0,0001**
		Domain 2	**0,46**	**0,09**	**0,0001**
		Domain 3	**0.32**	**0.06**	**0.0001**
		Domain 4	**0.56**	**0.12**	**0.0001**
Mild/no HME	546	Question 1	**0.07**	**0.03**	**0.0073**
		Question 2	0.03	0.03	0.2037
		Domain 1	**0.49**	**0.13**	**0.0003**
		Domain 2	**0.37**	**0.11**	**0.0007**
		Domain 3	**0.17**	**0.06**	**0.0105**
		Domain 4	**0.31**	**0,12**	**0,0125**
No HME	140	Question 1	0.05	0.05	0.2750
		Question 2	0.02	0.04	0.6437
		Domain 1	0.40	0.23	0.0789
		Domain 2	0.35	0.20	0.0719
		Question 3	0.11	0.11	0.2985
		Question 4	0.21	0.21	0.3262

Moderate HME, serum Mg ≥ 0.5 mmol/L and < 0.66 mmol/L; Moderate/Mild HME, serum Mg ≥ 0.5 mmol/L and < 0.8 mmol/L; Mild/No HME, serum Mg ≥ 0.66 mmol/L and < 0.8 mmol/L; No HME, serum Mg ≥ 0.8 mmol/L; severe HME cases (Mg < 0.5 mmol/L) were not included in the analysis due to limited sample size. HME, hypomagnesemia; Mg, magnesium; Δ_WHOQOL−BREF_, difference in World Health Organization quality of life questionnaire scores; ΔMg_0.1_, difference in the total serum Mg level between Visit 1 and Visit 2 normalized by 0.1 mmol/L; p values reflect whether Δ_WHOQOL−BREF_ domains/questions are significantly and linearly related to the change in serum Mg level;

significant p values (<0.05) are presented in bold.

### Factors independently related to the normalization of serum Mg level

Connective tissue dysplasia was the only factor that was predictive of the normalization of total serum Mg at both cut-off values in the pregnant women cohort. Connective tissue dysplasia, absence of multivitamin supplements, and oedema had the highest level of statistical significance (p < 0.003).

In the women in the hormone-related conditions cohort, factors predicting normalization of serum Mg concentration at both cut-off values were the absence of the following Mg deficiency symptoms: tinnitus, tachycardia, suffocation, hair loss, brittleness of the nails, chronic stress, paresthesia, tremor and Chvostek’s sign (p < 0.05). Additional factors for the normalization of Mg at both cut-off values are shown in the Supplementary Information.

## Discussion and conclusions

The present analysis demonstrated that a four-week treatment with Mg-vitamin B_6_ combination in women with HME provided a high response rate of normalization of serum Mg concentration, and was associated with improvements in the severity of symptoms in pregnant women and quality of life score in women with hormone-related conditions. To date this work is one of the largest and most comprehensive on Mg deficiency in women. The study encompasses research questions related to the clinical significance of HME and treatment with Magne B_6_ or Magne B_6_ Forte in real-world clinical practice. The studied cohorts included pregnant women and women with hormone-related conditions from large (over 1 million people) and smaller cities across the Russian Federation. The generalizability of the results using such a wide geographical coverage and inclusion of varying size cities reduces the bias related to lifestyle and environmental conditions.

A high prevalence of HME was noted in 79% of pregnant women and 55% of women with hormone-related conditions when using the cut-off value of < 0.8 mmol/L. The high prevalence of HME in the Russian Federation could possibly be explained by an unbalanced diet, the consumption of types of food that increase Mg requirement (such as soft drinks containing phosphoric acid), and an overall decline in the micronutrient density of foods ^22^. Calcium (Ca) and Mg levels are closely related, both vary consistently throughout the menstrual cycle and women are sensitive to these variations^7^. Additionally, specific conditions, such as pregnancy, may lead to changes in the need for Mg. Previous studies showed that serum Mg level significantly drops after 18 weeks of gestation compared with measurements before this time^23^ and various studies in animal models have demonstrated that a normal gestation is associated with a higher intake of Mg^24^.

Treatment of Mg deficiency with the MgB_6_ combination for approximately four weeks provided a high response rate in terms of normalization of total serum Mg concentration. Pregnant women with HME had a very high response rate and serum Mg level was normalized to ≥ 0.66 mmol/L in 92.2% of women, and to ≥ 0.80 mmol/L in 73.8%. Women with hormone-related conditions showed a lower response rate (≥ 0.66 mmol/L: 78.4%; ≥ 0.80 mmol/L: 58.9%). An increase of 0.1 mmol/L in total serum Mg level was associated with an improvement in the severity of threatened miscarriage symptoms in pregnant women and in QoL in women with hormone-related conditions, particularly in the subgroups of moderate/mild HME women with total serum Mg level ≥ 0.5 mmol/L and < 0.8 mmol/L compared with serum Mg ≥ 0.5 mmol/L and < 0.66 mmol/L (moderate HME). Based on patient-reported outcomes, the cut-off value of 0.8 mmol/L showed a significant benefit compared with 0.66 mmol/L. HME is well-known to be associated with a number of chronic diseases (including diabetes, hypertension, coronary heart disease, and osteoporosis)^2, 25^, with clinical manifestations seen at very low Mg levels, potentially explaining the use of 0.66 mmol/L as cut-off value in clinical practice. However, non-specific symptoms may start to appear at serum Mg values between 0.66–0.8 mmol/L, but be underestimated or ignored by the patients. The results obtained in this analysis support this hypothesis: women with baseline serum Mg concentration between 0.66–0.8 mmol/L showed a significant improvement in their quality of life after one month of Mg supplementation. Noteworthy, this change was observed neither in the subgroup of women with baseline serum Mg < 0.66 mml/L nor in the subgroup of women with Mg levels ≥ 0.8 mmol/L, further supporting the hypothesis that patients suffering from mild hypomagnesemia (below 0.8 mmol/L) are the ones who could benefit the most from Mg supplementation, whereas those with moderate hypomagnesemia (below 0.66 mmol/L) may require additional treatment to tackle the primary cause of Mg deficiency.

Many factors were found to be related to the normalization of Mg level in both cohorts. Some of them were unexpected and need further clarification in subsequent studies. Overall, traits associated with Mg level normalization at both cut-off values were related to the absence of Mg deficiency symptoms, cardiovascular and thyroid disorders. The absence of multivitamin supplementation was associated with normalization of total serum Mg concentration to ≥ 0.8 mmol/L, in both study cohorts. This concordance in results provides evidence that the observed effect is reliable, although the mechanistic basis of this association (as well as elucidating whether it is primary or secondary to other factors) still needs further clarification. It has been shown that changes in the intake of Mg may affect the metabolism of other minerals, such as Ca, and vice-versa^26^. Additionally, Ca has been proven to compete with Mg for intestinal absorption, thus potentially reducing the final intake of Mg^27^. In light of these findings, it could be hypothesized that the intake of multivitamins, usually containing also Ca, might have affected the results.

This analysis presents a few limitations. Diet or lifestyle changes that might have influenced Mg levels were not controlled in the observational studies, and there was no control arm. Another limitation is that data generated using the pooled databases cannot be generalized to the entire population of pregnant women or women with hormone-related conditions in the Russian Federation since those studies recruited only women with symptoms of Mg deficiency. Moreover, all women may not have received their four-week treatment before serum Mg was measured (or vice versa). The short follow-up did not allow collecting valuable data regarding childbirth conditions in pregnant women receiving Mg supplementation. Concomitant causes for non-HME specific symptoms have not been considered during the study. Lastly, the retrospective observational design of this study did not allow causal inferences to be made; however, it provided reliable data in a real-world setting, on the association between Mg supplementation and improvement in the symptoms and QoL in groups of women who most frequently suffer Mg deficiency.

To conclude, a high proportion of women with Mg deficiency had Mg level normalized after 1 month of treatment with Magne B_6_/Magne B_6_ Forte, regardless of the serum Mg cut-off value. Increasing total serum Mg level by 0.1 mmol/L was correlated with changes in various clinical/laboratory traits, reduction of the number of complaints, threatened miscarriage symptoms in pregnant women, and improvement of QoL, in < 0.8 mmol/L cut-off. These results allow us to support further serum Mg < 0.8 mmol/L as the most clinically relevant margin and encourage scientific community to conduct other studies. Among the factors associated with better treatment response, it is interesting to note that the use of multivitamins may interfere with the response to Mg supplementation, possibly related to the antagonism of Ca, however, further studies are needed to elucidate this finding.

## Supplementary Information

Supplementary information.

## Data Availability

Data may be provided upon reasonable request.

## References

[1] Ismail AAA, Ismail NA (2016). Magnesium: A mineral essential for health yet generally underestimated or even ignored. J. Nutr. Food Sci..

[2] Swaminathan R (2003). Magnesium metabolism and its disorders. Clin. Biochem. Rev..

[3] Yee J (2018). Magnesium: An important orphan. Adv. Chronic Kidney Dis..

[4] Workinger JL, Doyle RP, Bortz J (2018). Challenges in the diagnosis of magnesium status. Nutrients.

[5] Rude RK (1998). Magnesium deficiency: A cause of heterogenous disease in humans. J. Bone Mineral Res..

[6] Zarean E, Tarjan A (2017). Effect of magnesium supplement on pregnancy outcomes: A randomized control trial. Adv. Biomed. Res..

[7] Tonick S, Muneyyirci-Delale O (2016). Magnesium in women’s health and gynecology. Open J. Obstet. Gynecol..

[8] Spätling L, Classen HG, Külpmann WR, Manz F, Rob PM, Schimatschek HF, Vierling W, Vormann J, Weigert A, Wink K (2000). Diagnosing magnesium deficiency. Current recommendations of the society for magnesium research. Fortschr. Med. Orig..

[9] Lowenstein FW, Stanton MF (1986). Serum magnesium levels in the United States, 1971–1974. J. Am. Coll. Nutr..

[10] Liebscher D-H, Liebscher D-E (2004). About the misdiagnosis of magnesium deficiency. J. Am. Coll. Nutr..

[11] Bell CA (1995). Clinical guide to laboratory tests. Transfusion.

[12] Elin RJ (2010). Assessment of magnesium status for diagnosis and therapy. Magnes. Res..

[13] Costello RB, Elin RJ, Rosanoff A, Wallace TC, Guerrero-Romero F, Hruby A, Lutsey PL, Nielsen FH, Rodriguez-Moran M, Song Y, Van Horn LV (2016). Perspective: The case for an evidence-based reference interval for serum magnesium: the time has come. Adv. Nutr..

[14] Makatsariya AD, Bitsadze VO, Khizroeva DK, Dzhobava EM (2012). Prevalence of magnesium deficiency in pregnant women. Voprosy ginekologii, akusherstva i perinatologii.

[15] Serov VN, Blinov DV, Zimovina UV, Dzhobava EM (2014). Results of an investigation of the prevalence of magnesium deficiency in pregnant women. Akush. Ginekol..

[16] Serov VN, Baranov II, Blinov DV, Zimovina UV, Sandakova EA, Ushakova TI (2015). Results of evaluating Mg deficiency among female patients with hormone-related conditions. Akush. Ginekol..

[17] Makatsariya AD, Dadak C, Bitsadze VO, Solopova AG, Khamani NM (2017). Clinical features of patients with hormone-dependent conditions and magnesium deficiency. Akush. Ginekol..

[18] Orlova S, Dikke G, Pickering G, Konchits S, Starostin K, Bevz A (2020). Magnesium deficiency questionnaire: A new non-invasive magnesium deficiency screening tool developed using real-world data from four observational studies. Nutrients.

[19] Orlova, S., Dikke, G., Pickering, G., Yaltseva, N., Konchits, S., Starostin, K. & Bevz, A. (2020). Risk factors and comorbidities associated with magnesium deficiency in pregnant women and women with hormone-related conditions: Analysis of a large real-world dataset. Manuscript is submitted to BMC Pregnancy and Childbirth and available as a preprint. 10.21203/rs.3.rs-48032/v1. 10.1186/s12884-021-03558-2 PMC7821493 33482760

[20] World Health Organization WHOQOL-BREF Introduction. administration, scoring and generic version of the assessment. Field Trial Version. Programme on mental health, Geneva.

[21] Pham P-CT, Pham P-AT, Pham SV, Pham P-TT, Pham P-MT, Pham P-TT (2014). Hypomagnesemia: A clinical perspective. Int. J. Nephrol. Renovasc. Dis..

[22] DiNicolantonio JJ, O'Keefe JH, Wilson W (2018). Subclinical magnesium deficiency: A principal driver of cardiovascular disease and a public health crisis. Open Heart.

[23] Arikan G, Guecer F, Schoell W, Weiss P (1997). Preterm labour during oral magnesium supplementation in uncomplicated pregnancies. Geburtshilfe Frauenheilkd.

[24] Spatling L, Classen H, Kisters K, Liebscher U, Rylander R, Vierling W, Ehrlich B, Vormann J (2017). Supplementation of magnesium in pregnancy. J. Pregnancy Child Health.

[25] Van Laecke S (2019). Hypomagnesemia and hypermagnesemia. Acta Clin. Belg..

[26] Bertinato J, Preedy V, Patel VB (2017). Magnesium deficiency: Prevalence, assessment, and physiological effects. Handbook of Famine, Starvation, and Nutrient Deprivation: From Biology to Policy.

[27] Dai Q, Shu X-O, Deng X, Xiang Y-B, Li H, Yang G, Shrubsole MJ, Ji B, Cai H, Chow W-H, Gao Y-T, Zheng W (2013). Modifying effect of calcium/magnesium intake ratio and mortality: A population-based cohort study. BMJ Open.

