# Supplementary material for: Magnesium level correlation with clinical status and quality of life in women with hormone related conditions and pregnancy based on real world data
Source: Sci Rep. 2021 Mar 11;11:5734. doi: 10.1038/s41598-021-85156-y (PMC7952720; doi:10.1038/s41598-021-85156-y)
Supplement: Supplementary file 1 — Supplementary information. [file 41598_2021_85156_MOESM1_ESM.docx]

# SUPPLEMENTARY MATERIAL

**Supplementary material 1**

WHOQOL−BREF consists of 26 questions.[[20](#_ENREF_20)] Question 1 and Question 2 are general (“How would you rate your quality of life?” and “How satisfied are you with your health?”).

The remaining 24 questions are grouped in 4 domains related to different aspects of quality of life (**Table A1**).

Domain scores are scaled in a positive direction (i.e., higher scores denote higher quality of life). The mean score of items within each domain is used to calculate the domain score.

**Table A1**. WHOQOL−BREF domains

| **Domain** | **Facets incorporated within domains** |
| --- | --- |
| Physical health | Activities of daily living (Q17)  Dependence on medicinal substances and medical aids (Q4)  Energy and fatigue (Q10)  Mobility (Q15)  Pain and discomfort (Q3)  Sleep and rest (Q16)  Work capacity (Q18) |
| Psychological | Bodily image and appearance (Q11)  Negative feelings (Q26)  Positive feelings (Q5)  Self-esteem (Q6)  Spirituality / Religion / Personal beliefs (Q19)  Thinking, learning, memory and concentration (Q7) |
| Social relationships | Personal relationships (Q20)  Social support (Q21)  Sexual activity (Q22) |
| Environment | Financial resources (Q12)  Freedom, physical safety and security (Q8)  Home environment (Q23)  Health and social care: accessibility and quality of home environment (Q24)  Opportunities for acquiring new information and skills (Q13)  Participation in and opportunities for recreation / leisure activities (Q14)  Physical environment (pollution / noise / traffic / climate) (Q9)  Transport (Q25) |

The score for each question range from a minimum of 1 to a maximum of 5 points. Participants are required to answer all the questions. The following formulas are used to calculate the final questionnaire.

- Domain 1. Physical health – (6-Q3)+(6-Q4)+(Q10+Q15+Q16+Q17+Q18)
- Domain 2. Psychological health – (Q5+Q6+Q7+Q11+Q19)+(6–Q26)
- Domain 3. Social relationships – (Q20+Q21+Q22)
- Domain 4. Environment (social well-being) – (Q8+Q9+Q12+Q13+Q14+Q23+Q24+Q25)
- **Supplementary material 2**

**Table A2.** Factors that predict normalization of serum magnesium concentration in response to magnesium supplementation in pregnant women with symptoms of magnesium deficiency.

|  | **Cut-off <0.66 mmol/L** | | | | | **Cut-off <0.8 mmol/L** | | | |
| --- | --- | --- | --- | --- | --- | --- | --- | --- | --- |
|  | **N** | **OR^a^** | **95% CI** | | ***p-value*** | **N** | **OR^a^** | **95% CI** | ***p-value*** |
| **Age, years** | 318 | 0.986 | 0.916−1.062 | | 0.7070 | 717 | 0.984 | 0.954−1.015 | 0.3105 |
| **BMI, kg/m2** | 318 | 0.996 | 0.917−1.082 | | 0.9191 | 717 | 0.975 | 0.942−1.009 | 0.1473 |
| **Systolic blood pressure, mmHg** | 318 | 1.001 | 0.964−1.039 | | 0.9752 | 716 | 1.002 | 0.987−1.017 | 0.7740 |
| **Diastolic blood pressure, mmHg** | 318 | 1.009 | 0.965−1.054 | | 0.6995 | 716 | 1.003 | 0.984−1.022 | 0.7643 |
| **Heart rate, bpm** | 318 | 1.073 | 0.993−1.160 | | 0.0758 | 716 | 0.974 | 0.953−0.996 | **0.0226** |
| **Hospitalization Yes vs No** | 318 | 0.654 | 0.183−2.345 | | 0.5148 | 717 | 0.986 | 0.559−1.737 | 0.9606 |
| **Pregnancy number 2 vs 1** | 317 | 0.842 | 0.301−2.358 | | 0.7439 | 714 | 1.072 | 0.701−1.641 | 0.7477 |
| **Pregnancy number 3 vs 1** | 317 | 0.950 | 0.279−3.233 | | 0.9351 | 714 | 1.062 | 0.657−1.716 | 0.8063 |
| **Pregnancy number 2-3 vs 1** | 318 | 0.882 | 0.353−2.205 | | 0.7877 | 717 | 1.068 | 0.740−1.543 | 0.7256 |
| **Pregnancy number >3 vs 1** | 318 | 0.807 | 0.258−2.526 | | 0.7125 | 717 | 0.999 | 0.623−1.603 | 0.9981 |
| **Past gynaecological diseases, Yes vs No** | 318 | 0.767 | 0.278−2.119 | | 0.6094 | 717 | 1.024 | 0.686−1.528 | 0.9065 |
| Dysmenorrhea, Yes vs No | 318 | 1.074 | 0.387−2.975 | | 0.8914 | 717 | 1.072 | 0.710−1.619 | 0.7402 |
| Endometriosis, Yes vs No | 318 | 0.797 | 0.175−3.638 | | 0.7699 | 717 | 0.820 | 0.408−1.647 | 0.5765 |
| Malformations^b^, Yes vs No | 318 | n/a | n/a | | n/a | 717 | 0.708 | 0.129−3.900 | 0.6921 |
| STI, Yes vs No | 318 | 0.801 | 0.341−1.880 | | 0.6100 | 717 | 1.095 | 0.760−1.580 | 0.6257 |
| Uterine fibroids, Yes vs No | 318 | 0.525 | 0.185−1.490 | | 0.2261 | 717 | 1.172 | 0.652−2.107 | 0.5962 |
| Ovarian cysts, Yes vs No | 318 | 0.684 | 0.191−2.446 | | 0.5591 | 717 | 1.004 | 0.562−1.793 | 0.9898 |
| Ovarian cystoma | 318 | n/a | n/a | | n/a | 717 | 0.117 | 0.012−1.130 | 0.0637 |
| Cervical diseases, Yes vs No | 318 | 0.869 | 0.349−2.163 | | 0.7626 | 717 | 1.228 | 0.826−1.828 | 0.3103 |
| Ectopia cylindrical epithelium Yes vs No | 318 | 1.404 | 0.542−3.632 | | 0.4846 | 717 | 0.958 | 0.670−1.371 | 0.8159 |
| Leukoplakia | 318 | n/a | n/a | | n/a | 717 | 1.065 | 0.110−10.301 | 0.9563 |
| Diseases vulva, Yes vs No | 318 | 0.332 | 0.036−3.091 | | 0.3328 | 717 | 0.796 | 0.242−2.616 | 0.7072 |
| Endocrine disorders, Yes vs No | 318 | 0.666 | 0.215−2.061 | | 0.4809 | 717 | 0.969 | 0.523−1.794 | 0.9197 |
| Hyperprolactinemia, Yes vs No | 318 | 0.416 | 0.047−3.709 | | 0.4324 | 717 | 0.209 | 0.049−0.882 | **0.0331** |
| Abnormal uterine bleeding | 318 | n/a | n/a | | n/a | 717 | 1.188 | 0.323−4.364 | 0.7951 |
| Other | 318 | n/a | n/a | | n/a | 717 | 1.245 | 0.623−2.489 | 0.5348 |
| **Pregnancy complication^c^, Yes vs No** | 318 | 1.233 | 0.527−2.883 | | 0.6286 | 717 | 1.157 | 0.811−1.650 | 0.4209 |
| Gestational diabetes | 318 | n/a | n/a | | n/a | 717 | 1.424 | 0.158−12.823 | 0.7524 |
| Antiphospholipid syndrome, Yes vs No | 318 | 0.248 | 0.025−2.479 | | 0.2354 | 717 | 0.087 | 0.010−0.785 | **0.0295** |
| Placental abruption, Yes vs No | 318 | 0.332 | 0.036−3.091 | | 0.3328 | 717 | 1.247 | 0.257−6.057 | 0.7842 |
| Preeclampsia, Yes vs No | 318 | 1.296 | 0.372−4.513 | | 0.6842 | 717 | 1.714 | 0.985−2.982 | 0.0567 |
| Foetal death, Yes vs No | 318 | 0.119 | 0.019−0.748 | | **0.0233** | 717 | 0.707 | 0.175−2.857 | 0.6269 |
| Thrombotic complications, Yes vs No | 318 | 0.240 | 0.046−1.259 | | 0.0915 | 717 | 0.389 | 0.148−1.024 | 0.0560 |
| Foetal loss syndrome, Yes vs No | 318 | 0.119 | 0.019−0.748 | | **0.0233** | 717 | 0.513 | 0.256−1.032 | 0.0612 |
| Bleeding postpartum period | 318 | 0.240 | 0.046−1.259 | | 0.0915 | 717 | 1.159 | 0.373−3.599 | 0.7987 |
| Placental insufficiency, Yes vs No | 318 | 0.283 | 0.086−0.930 | | **0.0376** | 717 | 1.185 | 0.752−1.866 | 0.4644 |
| Intrauterine growth retardation, Yes vs No | 318 | n/a | n/a | | n/a | 717 | 0.838 | 0.361−1.948 | 0.6817 |
| Other | 318 | 0.903 | 0.346−2.354 | | 0.8343 | 717 | 1.379 | 0.553−3.439 | 0.4913 |
| **Comorbidities (general history), Yes vs No** | 318 | 1.385 | 0.176−10.895 | | 0.7567 | 717 | 2.074 | 1.221−3.521 | **0.0069** |
| Hypothyroidism, Yes vs No | 318 | n/a | n/a | | n/a | 717 | 0.619 | 0.361−1.059 | 0.0802 |
| Hepatitis, Yes vs No | 318 | 1.462 | 0.409−5.226 | | 0.5591 | 717 | 1.028 | 0.473−2.235 | 0.9447 |
| Asthenic syndrome, Yes vs No | 318 | 0.899 | 0.255−3.173 | | 0.8687 | 717 | 1.601 | 1.013−2.530 | **0.0440** |
| Viral infections, Yes vs No | 318 | 0.907 | 0.387−2.126 | | 0.8227 | 717 | 1.116 | 0.775−1.605 | 0.5557 |
| Connective tissue dysplasia^d^, Yes vs No | 318 | 2.781 | 1.216−6.360 | | **0.0154** | 717 | 1.728 | 1.230−2.428 | **0.0016** |
| Other, Yes vs No | 318 | 0.297 | 0.108−0.812 | | **0.0180** | 717 | 0.851 | 0.609−1.190 | 0.3466 |
| Multivitamin supplements, Yes vs No | 318 | 1.122 | 0.453−2.781 | | 0.8040 | 717 | 0.584 | 0.411−0.828 | **0.0026** |
| Threatened miscarriage^e^, Yes vs No | 318 | 0.739 | 0.299−1.829 | | 0.5134 |  | 1.273 | 0.902−1.797 | 0.1695 |
| **Complaints** |  |  |  |  |  |  |  |  |  |
| Oedema, Yes vs No | 318 | 1.187 | 0.495−2.843 | | 0.7008 | 717 | 2.443 | 1.611−3.705 | **<0.0001** |
| Lower abdominal discomfort, Yes vs No | 318 | 1.239 | 0.497−3.088 | | 0.6457 | 717 | 1.669 | 1.154−2.415 | **0.0066** |
| Spotting, Yes vs No | 318 | 2.635 | 0.344−20.191 | | 0.3511 | 717 | 1.212 | 0.651−2.256 | 0.5444 |
| Tetania gravidarum, Yes vs No | 318 | 1.641 | 0.635−4.239 | | 0.3065 | 717 | 1.104 | 0.764−1.596 | 0.5966 |
| Pelvic girdle pain, Yes vs No | 318 | 1.773 | 0.645−4.874 | | 0.2667 | 712 | 1.505 | 0.997−2.271 | 0.0518 |
| **Urine test abnormal** | 316 | n/a | n/a | | n/a | 715 | 0.700 | 0.127−3.852 | 0.6816 |
| **Platelets, 10^9^/L** | 317 | 0.999 | 0.993−1.005 | | 0.7588 | 704 | 0.996 | 0.993−0.999 | **0.0058** |
| **Erythrocyte sedimentation rate, mm/h** | 317 | 1.015 | 0.974−1.057 | | 0.4910 | 642 | 0.998 | 0.982−1.015 | 0.8494 |
| **ALT, U/L** | 291 | 0.992 | 0.954−1.031 | | 0.6823 | 639 | 0.988 | 0.973−1.004 | 0.1480 |
| **AST, U/L** | 290 | 1.023 | 0.970−1.079 | | 0.4071 | 709 | 1.017 | 0.997−1.037 | 0.0972 |
| **Total protein, g/L** | 314 | 1.005 | 0.963−1.049 | | 0.8160 | 717 | 1.008 | 0.987−1.030 | 0.4365 |

^a^Estimated using logistic regresssion. ^b^Malformations of the uterus, vagina. ^c^In past medical history. ^d^Asthenic body type; scoliosis; flat feet; myopia; valvular insufficiency; vegetative dystonia; or other signs. ^e^Diagnosed as having at least one of the following conditions: myometrium hypertonus; chorion detachment; preeclampsia; placental insufficiency; gestosis.

N, number of patients included in the analysis; ALT, alanine aminotransferase; AST, aspartate aminotransferase; BMI, body mass index; CI, confidence interval; OR, odds ratio; STI, sexually transmitted infection; p values of linear regression model reflect whether factors being tested are significantly and linearly related to serum Mg level normalization.

**Table A3.** Factors that predict normalization of serum magnesium concentration in response to magnesium supplementation in women with hormone-related conditions with mild hypomagnesemia.

|  | **Cut-off <0.66 mmol/L** | | | | **Cut-off <0.8 mmol/L** | | | |
| --- | --- | --- | --- | --- | --- | --- | --- | --- |
|  | **N** | **OR^a^** | **95% CI** | ***p-value*** | **N** | **OR^a^** | **95% CI** | ***p-value*** |
| **Age, years** | 361 | 1.001 | 0.980−1.023 | 0.9041 | 767 | 0.998 | 0.986−1.010 | 0.7635 |
| **BMI, kg/m2** | 360 | 0.973 | 0.925−1.023 | 0.2843 | 764 | 0.994 | 0.967−1.022 | 0.6811 |
| **Hormonal contraception^b^, Yes vs No** | 361 | 0.591 | 0.314−1.112 | 0.1031 | 767 | 1.063 | 0.730−1.548 | 0.7498 |
| **Premenstrual syndrome^b^, Yes vs No** | 361 | 0.872 | 0.466−1.632 | 0.6691 | 767 | 0.931 | 0.657−1.320 | 0.6886 |
| **Climacteric syndrome without HRT^c^, Yes vs No** | 361 | 1.230 | 0.645−2.344 | 0.5292 | 767 | 1.022 | 0.695−1.504 | 0.9110 |
| **HRT^c^, Yes vs No** | 361 | 0.879 | 0.463−1.671 | 0.6949 | 767 | 0.750 | 0.513−1.096 | 0.1373 |
| **Osteoporosis^b^, Yes vs No** | 361 | 1.964 | 0.982−3.927 | 0.0563 | 767 | 0.928 | 0.652−1.321 | 0.6781 |
| **Other hormonal conditions^d^, Yes vs No** | 361 | 0.832 | 0.443−1.559 | 0.5653 | 767 | 0.935 | 0.652−1.340 | 0.7131 |
| **Multivitamin supplements, Yes vs No** | 361 | 1.051 | 0.598−1.847 | 0.8624 | 767 | 0.705 | 0.515−0.964 | **0.0287** |
| **Oral Combined Contraceptives, type 2-phase vs 1-phase** | 57 | 0.784 | 0.195−3.155 | 0.7325 | 128 | 0.339 | 0.124−0.923 | **0.0343** |
| **Oral Combined Contraceptives, type 3-phase vs 1-phase** | 57 | 1.345 | 0.128−14.181 | 0.8054 | 128 | 0.252 | 0.047−1.361 | 0.1092 |
| **Magnesium deficiency symptoms** |  |  |  |  |  |  |  |  |
| Weakness | 309 | 0.953 | 0.860−1.056 | 0.3553 | 652 | 0.903 | 0.847−0.962 | **0.0016** |
| Sleep disturbance | 309 | 0.876 | 0.791−0.969 | **0.0105** | 607 | 0.951 | 0.895−1.010 | 0.1023 |
| Tinnitus | 156 | 0.657 | 0.538−0.800 | **<0.0001** | 322 | 0.755 | 0.673−0.846 | **<0.0001** |
| Tachycardia | 202 | 0.865 | 0.763−0.980 | **0.0227** | 408 | 0.907 | 0.840−0.978 | **0.0116** |
| Hot flash | 175 | 0.906 | 0.799−1.027 | 0.1235 | 356 | 0.877 | 0.818−0.941 | **0.0002** |
| Excessive sweating | 209 | 0.882 | 0.776−1.004 | 0.0571 | 436 | 0.893 | 0.831−0.961 | **0.0023** |
| Suffocation | 113 | 0.825 | 0.683−0.997 | **0.0464** | 265 | 0.839 | 0.747−0.961 | **0.0029** |
| Numbness of limbs | 213 | 0.876 | 0.760−1.010 | 0.0684 | 458 | 0.947 | 0.871−1.030 | 0.2030 |
| Irritability | 336 | 0.941 | 0.852−1.040 | 0.2360 | 687 | 0.974 | 0.920−1.031 | 0.3693 |
| Hair loss, brittleness of the nails | 261 | 0.878 | 0.782−0.986 | **0.0283** | 559 | 0.902 | 0.842−0.966 | **0.0031** |
| Convulsions of lower limbs | 217 | 1.001 | 0.852−1.175 | 0.9940 | 491 | 0.950 | 0.882−1.022 | 0.1687 |
| Sleep disorders | 296 | 0.903 | 0.817−0.998 | **0.0456** | 600 | 0.969 | 0.913−1.028 | 0.2892 |
| Chronic stress | 280 | 0.897 | 0.816−0.986 | **0.0242** | 567 | 0.943 | 0.892−0.997 | **0.0397** |
| Muscle weakness | 254 | 1.027 | 0.910−1.159 | 0.6614 | 543 | 0.912 | 0.851−0.978 | **0.0095** |
| Feeling a lump in the throat | 126 | 0.850 | 0.682−1.061 | 0.1506 | 281 | 0.910 | 0.812−1.020 | 0.1054 |
| Stomach ache, abdominal cramps | 150 | 0.956 | 0.817−1.118 | 0.5699 | 350 | 0.855 | 0.787−0.930 | **0.0003** |
| Back pain | 224 | 0.920 | 0.825−1.026 | 0.1339 | 467 | 0.945 | 0.883−1.010 | 0.0969 |
| Dizziness | 211 | 0.885 | 0.767−1.021 | 0.0950 | 433 | 0.836 | 0.766−0.912 | **0.0001** |
| Paraesthesia | 166 | 0.815 | 0.706−0.942 | **0.0056** | 336 | 0.840 | 0.761−0.927 | **0.0005** |
| Fast fatigability | 304 | 1.011 | 0.910−1.123 | 0.8406 | 650 | 1.015 | 0.956−1.078 | 0.6238 |
| Tremor | 119 | 0.743 | 0.618−0.894 | **0.0016** | 259 | 0.836 | 0.742−0.941 | **0.0032** |
| Frequent headaches | 237 | 0.969 | 0.859−1.094 | 0.6143 | 509 | 0.965 | 0.903−1.030 | 0.2850 |
| Chvostek's sign | 86 | 0.640 | 0.464−0.882 | 0.0063 | 202 | 0.668 | 0.538−0.829 | **0.0002** |
| **Clinical and laboratory tests performed within 28-30 days prior to enrolment (data from medical records)** |  |  |  |  |  |  |  |  |
| Abnormalities on ultrasound of the pelvis, Yes vs No | 323 | 1.024 | 0.582−1.799 | 0.9350 | 699 | 1.111 | 0.818−1.508 | 0.5016 |
| Abnormalities on ECG, Yes vs No | 230 | 0.650 | 0.326−1.296 | 0.2215 | 466 | 0.896 | 0.582−1.380 | 0.6178 |
| Haemoglobin, g/L | 325 | 1.018 | 0.996−1.040 | 0.1139 | 699 | 1.008 | 0.995−1.021 | 0.2321 |
| Platelets, 109/L | 314 | 1.003 | 0.998−1.008 | 0.2743 | 649 | 0.999 | 0.996−1.002 | 0.5179 |
| Erythrocyte sedimentation rate, mm/h | 322 | 0.996 | 0.951−1.042 | 0.8543 | 686 | 0.974 | 0.946−1.003 | 0.0816 |
| ALT, U/L | 263 | 0.986 | 0.962−1.010 | 0.2503 | 562 | 0.994 | 0.978−1.009 | 0.4328 |
| AST, U/L | 253 | 0.994 | 0.968−1.020 | 0.6475 | 553 | 0.993 | 0.975−1.012 | 0.4660 |
| Total protein, g/L | 257 | 1.009 | 0.973−1.047 | 0.6245 | 544 | 1.051 | 1.024−1.079 | **0.0002** |
| Glucose, mmol/L | 287 | 1.019 | 0.762−1.361 | 0.9009 | 633 | 1.028 | 0.855−1.235 | 0.7728 |
| Total bilirubin, μmol/L | 254 | 0.984 | 0.954−1.014 | 0.2843 | 549 | 0.996 | 0.976−1.017 | 0.7350 |
| Total cholesterol, mmol/L | 241 | 1.087 | 0.837−1.411 | 0.5311 | 527 | 1.017 | 0.870−1.189 | 0.8285 |
| Osteocalcin, hg/ml | 80 | 1.011 | 0.983−1.039 | 0.4470 | 163 | 1.013 | 0.992−1.034 | 0.2185 |
| FSH, mU/L | 174 | 1.001 | 0.990−1.012 | 0.8557 | 371 | 0.999 | 0.995−1.004 | 0.7814 |
| LH, mU/L | 156 | 0.998 | 0.980−1.016 | 0.8032 | 329 | 0.999 | 0.992−1.006 | 0.8190 |
| Estradiol, pg/ml | 131 | 1.004 | 0.998−1.010 | 0.1738 | 292 | 1.003 | 1.000−1.006 | **0.0359** |
| Progesterone, nmol/L | 132 | 0.980 | 0.959−1.001 | 0.0574 | 257 | 0.999 | 0.985− 1.014 | 0.9318 |
| Testosterone, nmol/L | 121 | 0.983 | 0.933−1.037 | 0.5351 | 253 | 1.039 | 0.988−1.091 | 0.1336 |
| SHBG, nmol/L | 79 | 1.000 | 0.988−1.012 | 0.9391 | 157 | 1.008 | 0.999−1.017 | 0.0768 |
| Calcitonin, pg/ml | 77 | 1.015 | 0.957−1.077 | 0.6138 | 147 | 0.937 | 0.892−0.984 | **0.0093** |
| Parathormone, pg/ml | 72 | 1.007 | 0.987−1.027 | 0.5025 | 140 | 1.003 | 0.989−1.018 | 0.6431 |
| **Comorbidities (general history)** |  |  |  |  |  |  |  |  |
| Frequent viral infections, Yes vs No | 361 | 0.809 | 0.457−1.434 | 0.4683 | 767 | 0.807 | 0.582−1.119 | 0.1979 |
| Allergic reactions, Yes vs No | 361 | 0.683 | 0.341−1.368 | 0.2815 | 767 | 0.932 | 0.628−1.383 | 0.7276 |
| Bronchial asthma, Yes vs No | 361 | 0.821 | 0.217−3.109 | 0.7717 | 767 | 0.853 | 0.404−1.798 | 0.6752 |
| Scoliosis, flat feet, Yes vs No | 361 | 1.994 | 0.752−5.282 | 0.1652 | 767 | 1.540 | 0.943−2.515 | 0.0845 |
| Myopia, Yes vs No | 361 | 1.015 | 0.577−1.784 | 0.9601 | 767 | 0.932 | 0.675−1.287 | 0.6698 |
| ENT diseases, Yes vs No | 361 | 0.614 | 0.346−1.090 | 0.0958 | 767 | 0.996 | 0.698−1.422 | 0.9840 |
| Anaemia, Yes vs No | 361 | 0.848 | 0.366−1.962 | 0.6998 | 767 | 0.836 | 0.524−1.332 | 0.4506 |
| Type 1 diabetes, Yes vs No | 361 | 0.825 | 0.085−8.043 | 0.8685 | 767 | 2.804 | 0.312−25.202 | 0.3575 |
| Type 2 diabetes, Yes vs No | 361 | 1.668 | 0.198−14.063 | 0.6382 | 767 | 2.485 | 0.810−7.622 | 0.1114 |
| Dyslipidaemia, Yes vs No | 361 | 1.683 | 0.369−7.681 | 0.5018 | 767 | 1.755 | 0.858−3.593 | 0.1237 |
| Obesity, Yes vs No | 361 | 0.860 | 0.415−1.782 | 0.6843 | 767 | 1.071 | 0.711−1.616 | 0.7418 |
| Varicose veins, Yes vs No | 361 | 0.934 | 0.484−1.800 | 0.8378 | 767 | 1.178 | 0.796−1.743 | 0.4120 |
| Thrombosis, Yes vs No | 361 | n/a | n/a | n/a | 767 | 0.695 | 0.139−3.465 | 0.6570 |
| Vegetative-vascular dystonia, Yes vs No | 361 | 1.598 | 0.859−2.972 | 0.1387 | 767 | 1.280 | 0.924−1.774 | 0.1372 |
| Asthenic syndrome, Yes vs No | 361 | n/a | n/a | n/a | 767 | 0.893 | 0.513−1.553 | 0.6880 |
| **GIT diseases, Yes vs No** | 361 | 1.315 | 0.782−2.210 | 0.3018 | 767 | 0.930 | 0.696−1.244 | 0.6249 |
| Hepatitis, Yes vs No | 361 | 1.392 | 0.299−6.486 | 0.6739 | 767 | 1.317 | 0.552−3.145 | 0.5350 |
| Cholelithiasis, Yes vs No | 361 | 1.796 | 0.674−4.785 | 0.2415 | 767 | 1.026 | 0.639−1.649 | 0.9147 |
| Other diseases of the liver and bile ducts, Yes vs No | 361 | 0.962 | 0.308−3.011 | 0.9476 | 767 | 0.924 | 0.515−1.659 | 0.7915 |
| Chronic gastritis, chronic gastroduodenitis, Yes vs No | 361 | 1.224 | 0.693−2.162 | 0.4858 | 767 | 0.985 | 0.723−1.342 | 0.9229 |
| Gastric and duodenal ulcer, Yes vs No | 361 | 0.634 | 0.160−2.509 | 0.5159 | 767 | 1.200 | 0.467−3.083 | 0.7049 |
| Other Yes, vs No | 361 | 0.273 | 0.017−4.416 | 0.3607 | 767 | 0.172 | 0.019−1.550 | 0.1167 |
| **Kidneys and urinary tract diseases, Yes vs No** | 361 | 1.026 | 0.593−1.777 | 0.9256 | 767 | 0.874 | 0.640−1.194 | 0.3970 |
| Chronic pyelonephritis, Yes vs No | 361 | 1.121 | 0.549−2.290 | 0.7533 | 767 | 1.045 | 0.705−1.551 | 0.8251 |
| Urolithiasis, Yes vs No | 361 | 0.552 | 0.239−1.273 | 0.1634 | 767 | 0.903 | 0.514−1.585 | 0.7212 |
| Cystitis, Yes vs No | 361 | 1.293 | 0.548−3.053 | 0.5577 | 767 | 0.664 | 0.426−1.037 | 0.0717 |
| Other, Yes vs No | 361 | n/a | n/a | n/a | 767 | n/a | n/a− | n/a |
| **Neurological disease, Yes vs No** | 361 | 1.350 | 0.773−2.359 | 0.2921 | 767 | 0.972 | 0.715−1.322 | 0.8568 |
| Osteochondrosis, Yes vs No | 361 | 1.350 | 0.773−2.359 | 0.2921 | 767 | 0.956 | 0.703−1.302 | 0.7774 |
| Stroke, Yes vs No | 361 | n/a | n/a | n/a | 767 | n/a | n/a | n/a |
| Other, Yes vs No | 361 | n/a | n/a | n/a | 767 | 0.347 | 0.031−3.843 | 0.3883 |
| **Cardiovascular disease, Yes vs No** | 361 | 0.768 | 0.443−1.331 | 0.3465 | 767 | 0.832 | 0.597−1.159 | 0.2775 |
| Atherosclerosis, Yes vs No | 361 | 1.536 | 0.333−7.074 | 0.5821 | 767 | 1.412 | 0.652−3.059 | 0.3818 |
| Arterial hypertension, hypertonic disease, Yes vs No | 361 | 0.808 | 0.436−1.495 | 0.4969 | 767 | 0.900 | 0.621−1.305 | 0.5788 |
| Heart rhythm disturbance, Yes vs No | 361 | 0.435 | 0.190−0.992 | **0.0478** | 767 | 0.893 | 0.513−1.553 | 0.6880 |
| Pathology of heart valves, Yes vs No | 361 | 0.370 | 0.114−1.201 | 0.0978 | 767 | 0.282 | 0.099−0.810 | **0.0187** |
| Coronary heart disease, myocardial infarction, chronic heart failure, Yes vs No | 361 | 0.270 | 0.037−1.952 | 0.1947 | 767 | 1.046 | 0.293−3.736 | 0.9451 |
| Other Yes vs No | 361 | n/a | n/a | n/a | 767 | n/a | n/a | n/a |
| **Thyroid diseases, Yes vs No** | 361 | 4.339 | 1.012−18.600 | **0.0482** | 767 | 0.727 | 0.456−1.160 | 0.1811 |
| Hypothyroidism, Yes vs No | 361 | 2.581 | 0.586−11.370 | 0.2102 | 767 | 0.550 | 0.310−0.973 | **0.0400** |
| Nodular goitre, Yes vs No | 361 | n/a | n/a | n/a | 767 | 1.146 | 0.534−2.461 | 0.7264 |
| Thyrotoxicosis, Yes vs No | 361 | n/a | n/a | n/a | 767 | n/a | n/a | n/a |
| Other, Yes vs No | 361 | n/a | n/a | n/a | 767 | n/a | n/a | n/a |
| **Malignant neoplasms Yes vs No** | 361 | n/a | n/a | n/a | 767 | 1.397 | 0.254−7.675 | 0.7003 |
| Stomach, Yes vs No | 361 | n/a | n/a | n/a | 767 | n/a | n/a | n/a |
| Lung, Yes vs No | 361 | n/a | n/a | n/a | 767 | n/a | n/a | n/a |
| Other localization, Yes vs No | 361 | n/a | n/a | n/a | 767 | n/a | n/a | n/a |
| Other, Yes vs No | 361 | n/a | n/a | n/a | 767 | 0.696 | 0.097−4.964 | 0.7173 |
| Other chronic diseases, Yes vs No | 361 | n/a | n/a | n/a | 767 | 0.472 | 0.199−1.118 | 0.0879 |
| **Obstetric and gynaecological past medical history and status** |  |  |  |  |  |  |  |  |
| Diseases of the cervix and vulva, Yes vs No | 361 | 1.053 | 0.549−2.020 | 0.8755 | 767 | 0.963 | 0.687−1.350 | 0.8284 |
| Genital infection, Yes vs No | 361 | 1.076 | 0.474−2.447 | 0.8605 | 767 | 0.759 | 0.507−1.137 | 0.1818 |
| Malformations of the genital organs, Yes vs No | 361 | 0.273 | 0.017−4.416 | 0.3607 | 767 | 2.095 | 0.217−20.221 | 0.5225 |
| Inflammatory diseases of the genital organs, Yes vs No | 361 | 0.811 | 0.432−1.523 | 0.5156 | 767 | 0.809 | 0.553−1.182 | 0.2729 |
| Dysmenorrhea, Yes vs No | 361 | 1.291 | 0.474−3.515 | 0.6167 | 767 | 0.715 | 0.437−1.170 | 0.1823 |
| **Menstrual disorders** |  |  |  |  |  |  |  |  |
| Amenorrhea, oligo-spanio-menorrhea, Yes vs No | 361 | 4.614 | 0.602−35.348 | 0.1410 | 767 | 1.424 | 0.767−2.642 | 0.2626 |
| Hyper-poly-menorrhea, abnormal uterine bleeding, Yes vs No | 361 | 0.848 | 0.366−1.962 | 0.6998 | 767 | 1.088 | 0.676−1.753 | 0.7273 |
| Premenstrual syndrome, Yes vs No | 361 | 0.853 | 0.422−1.724 | 0.6580 | 767 | 0.771 | 0.525−1.134 | 0.1862 |
| Regular menstruation, Yes vs No | 361 | 0.584 | 0.338−1.010 | 0.0543 | 767 | 0.728 | 0.534−0.992 | **0.0446** |
| Irregular menstruation, Yes vs No | 361 | 1.476 | 0.593−3.675 | 0.4033 | 767 | 1.487 | 0.954−2.319 | 0.0800 |
| Perimenopause, Yes vs No | 361 | 1.331 | 0.439−4.034 | 0.6129 | 767 | 1.069 | 0.570−2.006 | 0.8342 |
| Natural menopause, Yes vs No | 361 | 0.761 | 0.429−1.352 | 0.3522 | 767 | 0.724 | 0.507−1.033 | 0.0746 |
| Surgical menopause, Yes vs No | 361 | 2.317 | 0.679−7.905 | 0.1798 | 767 | 0.710 | 0.398−1.267 | 0.2466 |
| Climacteric syndrome, Yes vs No | 361 | 1.202 | 0.659−2.192 | 0.5490 | 767 | 0.712 | 0.504−1.006 | 0.0543 |
| Prolapse of genital organs, Yes vs No | 361 | 0.535 | 0.177−1.613 | 0.2666 | 767 | 0.966 | 0.518−1.802 | 0.9133 |
| Urinary incontinence, Yes vs No | 361 | n/a | n/a | n/a | 767 | 1.794 | 0.904−3.562 | 0.0946 |
| Hyperplastic processes of the endometrium, Yes vs No | 361 | 2.211 | 0.646−7.568 | 0.1410 | 767 | 1.036 | 0.621−1.726 | 0.8935 |
| Endometriosis, Yes vs No | 361 | 1.530 | 0.738−3.171 | 0.2527 | 767 | 0.900 | 0.625−1.296 | 0.5700 |
| Uterine myoma, Yes vs No | 361 | 0.770 | 0.415−1.429 | 0.4073 | 767 | 0.588 | 0.418−0.827 | **0.0022** |
| Hyperprolactinemia, Yes vs No | 361 | n/a | n/a | n/a | 767 | 0.868 | 0.231−3.259 | 0.8344 |
| Polycystic ovary syndrome, Yes vs No | 361 | n/a | n/a | n/a | 767 | 1.739 | 0.790−3.829 | 0.1694 |
| Ovarian cysts and cystomas, Yes vs No | 361 | 1.301 | 0.364−4.646 | 0.6854 | 767 | 0.854 | 0.443−1.646 | 0.6377 |
| Infertility, Yes vs No | 361 | 1.257 | 0.413−3.827 | 0.6878 | 767 | 0.880 | 0.493−1.568 | 0.6632 |
| **Complications of pregnancy and childbirth Yes vs No** | 361 | 1.273 | 0.690−2.349 | 0.4391 | 767 | 1.154 | 0.818−1.629 | 0.4139 |
| Ectopic pregnancy, Yes vs No | 361 | 0.727 | 0.188−2.808 | 0.6440 | 767 | 0.691 | 0.271−1.760 | 0.4382 |
| Syndrome of miscarriage of the foetus, Yes vs No | 361 | 0.748 | 0.232−2.417 | 0.6277 | 767 | 1.492 | 0.666−3.342 | 0.3310 |
| Threatened miscarriage, Yes vs No | 361 | 1.878 | 0.850−4.148 | 0.1193 | 767 | 1.279 | 0.853−1.917 | 0.2346 |
| Preeclampsia, Yes vs No | 361 | 0.646 | 0.221−1.893 | 0.4259 | 767 | 1.684 | 0.728−3.895 | 0.2236 |
| Fetoplacental insufficiency, Yes vs No | 361 | 1.291 | 0.474−3.515 | 0.6167 | 767 | 1.430 | 0.808−2.530 | 0.2193 |
| Foetal death, Yes vs No | 361 | 0.547 | 0.049−6.112 | 0.6244 | 767 | 1.397 | 0.254−7.675 | 0.7003 |
| Premature birth, Yes vs No | 361 | 0.607 | 0.182−2.027 | 0.4172 | 767 | 0.689 | 0.295−1.610 | 0.3901 |
| **Gynaecological and obstetric operations Yes vs No** | 361 | 1.320 | 0.633−2.752 | 0.4595 | 767 | 1.232 | 0.816−1.858 | 0.3207 |
| **Diseases of the breast Yes vs No** | 361 | 5.618 | 0.922−34.231 | 0.0612 | 767 | 1.441 | 0.358−5.803 | 0.6077 |
| Diffuse form of fibrocystic mastopathy, Yes vs No | 361 | 1.307 | 0.644−2.650 | 0.4580 | 767 | 0.833 | 0.581−1.193 | 0.3184 |
| Nodular form of fibrocystic mastopathy, Yes vs No | 361 | 0.268 | 0.053−1.354 | 0.1111 | 767 | 0.460 | 0.129−1.643 | 0.2318 |
| Fibroadenoma, Yes vs No | 361 | n/a | n/a | n/a | 767 | 1.399 | 0.347−5.636 | 0.6367 |
| Cancer, Yes vs No | 361 | n/a | n/a | n/a | 767 | 2.095 | 0.217−20.221 | 0.5225 |
| **Malignant neoplasms of the genital organs, Yes vs No** | 361 | n/a | n/a | n/a | 767 | 0.231 | 0.024−2.228 | 0.2050 |
| **Other gynaecological diseases, Yes vs No** | 361 | 1.011 | 0.275−3.717 | 0.9869 | 767 | 0.840 | 0.408−1.730 | 0.6367 |

^a^Estimated using logistic regression. ^b^ Disregarding an allocation at the enrolment in the original studies. ^c^ Including surgical menopause. ^d^ Women of reproductive age with other hormonal conditions: endometriosis; polycystic ovarian disease; uterine leiomyoma; algodismenorrhea; endometrial hyperplastic processes

N, number of patients included in the analysis; ALT, alanine aminotransferase; AST, aspartate aminotransferase; BMI, body mass index; CI, confidence interval; ECG, electrocardiogram; FSH, Follicle-stimulating hormone; HRT, hormone-replacement therapy; LH, Luteinizing hormone; OR, odds ratio; SHBG, Sex Hormone Binding Globulin; p values of linear regression model reflect whether factors being tested are significantly and linearly related to serum Mg level normalization.
